# Waste management optimization with NLP modeling and waste-to-energy in a circular economy

**DOI:** 10.1038/s41598-024-69321-7

**Published:** 2024-08-27

**Authors:** Ilse María Hernández-Romero, Javier Camilo Niño-Caballero, Lucy T. González, Michael Pérez-Rodríguez, Antonio Flores-Tlacuahuac, Alejandro Montesinos-Castellanos

**Affiliations:** 1 Tecnologico de Monterrey, Institute of Advanced Materials for Sustainable Manufacturing, Ave. Eugenio Garza Sada 2501, Monterrey, N.L. 64849 Mexico; 2 Tecnologico de Monterrey, Escuela de Ingenieria y Ciencias, Ave. Eugenio Garza Sada 2501, Monterrey, N.L. 64849 Mexico; 3 Tecnologico de Monterrey, Centro del Agua, Ave. Eugenio Garza Sada 2501, Monterrey, N.L. 64849 Mexico

**Keywords:** Municipal Solid Waste (MSW), Energy conversion, Waste- to-Energy, Multi-objective optimization, Social Carbon Cost (SCC), Decision-making framework, Mathematics and computing, Applied mathematics, Computational science

## Abstract

This work presents a methodology integrating Non-Linear Programming (NLP) for multi-objective and multi-period optimization, addressing sustainable waste management and energy conversion challenges. It integrates waste-to-energy (WtE) technologies such as Anaerobic Digestion (AD), Incineration (Inc), Gasification (Gsf), and Pyrolysis (Py), and considers thermochemical, technical, economic, and environmental considerations through rigorous non-linear functions. Using Mexico City as a case study, the model develops waste management strategies that balance environmental and economic aims, considering social impacts. A trade-off solution is proposed to address the conflict between objectives. The economical optimal solution generates 1.79M$ with 954 tons of CO_2_ emissions while the environmental one generates 0.91M$ and reduces emissions by 54%, where 40% is due to gasification technology. Moreover, the environmentally optimal solution, with incineration and gasification generates 9500 MWh/day and 5960 MWh/day, respectively, demonstrates the capacity of the model to support sustainable energy strategies. Finally, this work presents an adaptable framework for sustainable waste management decision-making.

## Introduction

In the last decades, increasing municipal solid waste (MSW) has emerged as a critical challenge in Latin America (LATAM)^1^. By 2050, MSW production is estimated to reach alarming figures, with projections of up to 3.40 billion tons per year, of which 70% will be unsustainable managed in landfills and open dumps^2^. These practices pose significant threats to the environment and public health in the region, such as groundwater contamination by leachates, sanitation problems for surrounding communities, and greenhouse gas emissions^3^.

Effective MSW management is essential to address this crisis and harness the potential of waste as a valuable resource, offering a viable solution for many countries, specifically in LATAM^4,5^. Waste-to-Energy (WtE) technologies emerge as a promising pathway to achieve sustainable regional waste management^6,7^. WtE processes reduce the volume of waste, replace fossil fuel-based power generation plants, and mitigate the release of harmful substances and pollutants. Consequently, WtE technologies has emerged as a global solution for sustainable waste management within a circular economy framework.

Waste-to-Energy technologies contribute to the circular economy primarily through the processing of biogenic waste, such as food and organic matter, which can be converted into energy and compost, thereby closing the nutrient loop. However, for non-biogenic wastes, like plastics (Pl), WtE serves as an energy recovery method rather than achieving full material circularity. While incineration of plastics reduces landfill volume and generates energy, it does not return materials to the production cycle, thus limiting its circularity. This integrated approach helps optimize waste management by balancing material recovery and energy generation.

Currently, over 2179 WtE facilities are operating worldwide, demonstrating its growing recognition and adoption in various regions. Moreover, the circular economy concept has gained traction as a key approach to tackling waste management challenges and promoting sustainability, not only in LATAM but globally^8–10^. In this regard, the circular economy seeks to close resource loops, promoting waste reuse, recycling, and energy recovery^11^. Implementing circular economy strategies in waste management can generate economic, environmental, and social benefits for the region^12^.

Several studies have addressed the need for transitions toward more sustainable practices in this context, where waste management faces particular challenges emphasizing the relevance of favorable policies and regulatory frameworks. For example^13^ developed a MSW management performance Index with 15 sustainability indicators. The index supports waste management in municipalities, aiding the implementation of sustainable strategies and policies. Its applicability is feasible in various countries, irrespective of municipality size. In the case of^14^, they critically reviewed waste management in LATAM, highlighting challenges and sustainable alternatives based on life cycle assessment to support environmentally responsible policies.

Additionally, studies have been conducted on the technical and economic feasibility of WtE technologies, such as^15^ they focus on competitive electricity generation using anaerobic energy conversion technologies (WtE-AD) in Mexico. Conducting environmental and economic assessments, they guide sustainable bioenergy investments. Similarly^16^ evaluates a proposed WtE plant in Havana, Cuba, demonstrating its feasibility. The plant could annually generate 227.1 GWh, meeting 6% of the demand, with a net present value of 35.48M USD.

Moreover, many studies do not consider the dynamic and variable nature of waste generation over time. Silva-Martínez et al. ^17^, explore the viability and environmental impact of WtE technologies. However, they don’t capture the seasonal variations and changing waste compositions that influence the performance of the WtE.

Additionally, previous works like^18^ have focused mainly on economic feasibility, with less emphasis on integrating detailed environmental and social metrics, such as the Social Cost of Carbon (SCC). Their study on waste incineration in Mexico found technical feasibility for populations up to 3 million, contributing 4.3% to national demand. They emphasize incineration as a renewable energy source, integral to economic development and regional job creation^18,19^.

In waste management systems^20,21^ employ simulation and optimization tools to design efficient systems. Mathematical programming, extensively used in optimal waste management design, is highlighted by^22,23^. Their findings underscore that while minimizing costs is a common objective, simultaneous consideration of environmental, economic, and social objectives is still lacking, especially in LATAM countries. Various mathematical and optimization models proposed by researchers like^24,25^ enhance waste management planning in LATAM. Diaz-Barriga-Fernandez et al.^26^ presents a multi-objective optimization strategy for municipal solid waste management, incorporating stakeholder interactions and supply chain dynamics. The study aims to optimize the system while addressing societal concerns according to^27^. Similarly^28^ proposes a model for optimal waste management planning, considering various waste types, locations, processing routes, and dynamic behaviors over time. Their model promotes sustainable policies and cooperation among cities.

On the other hand, NLP has been used in research to address waste-to-energy complexities through non-linear functions, optimizing technologies, and capturing dynamic relationships within the system using a robust mathematical framework. For instance^29^ optimized anaerobic digestion processes using a first-order kinetics model and a multi-objective NLP algorithm. Similarly^30^ used NLP to define refuse quantities and characteristics to be sent to different MSW treatment facilities to enhance waste management planning. Fiorucci et al. ^31^ employed NLP for technology selection focused on incineration, composting, anaerobic digestion, and landfilling, with the aim of minimizing total cost and carbon equivalent emissions. Lastly^32^ developed a mathematical model to optimize the MSW transportation system, minimizing waste transportation time and cost.

This work introduces a methodology integrating Non-Linear Programming (NLP) for multi-objective and multi-period optimization, addressing sustainable waste management and energy conversion challenges. The model incorporates various WtE technologies such as Anaerobic Digestion (AD), Incineration (Inc), Gasification (Gsf), and Pyrolysis (Py), considering thermochemical, technical, economic, environmental, and social aspects through rigorous non-linear functions. Using Mexico City as a case study, the model develops waste management strategies that balance environmental sustainability and economic objectives, considering social impacts and providing an approach to sustainable waste management within the circular economy framework.

Thus, this methodology also focuses on finding a compromise solution that seeks to maximize all the objectives simultaneously, recognizing that waste management decisions must balance economic benefits with environmental and social considerations. The role of the carbon cost is highlighted as a critical factor for process viability. Including the SCC in the economic evaluation helps quantify the benefits associated with greenhouse gas emissions reduction and provides a more comprehensive perspective on environmental impacts. Moreover, proximate and ultimate analysis of non-linear functions has been considered to determine the energy, heat, and other parameters of WtE conversion technologies^33^. Incorporating non-linear functions in the model makes it more robust and realistic, as it better captures the complexity and non-linearity associated with the waste conversion process.

In summary, the main contributions of this work are:

- This study combines different Waste-to-Energy (WtE) technologies, such as Anaerobic Digestion, Incineration, Gasification, and Pyrolysis, into a single optimization framework. This approach provides a more precise and realistic depiction of the potential synergies and trade-offs between these technologies.
- The model uses Non-Linear Programming (NLP) for multi-objective and multi-period optimization, taking into account economic, environmental, and social impacts simultaneously. This optimization approach tackles the inherent non-linearity of WtE processes.
- The study uses detailed and current data on waste composition and generation, including monthly variations and proximate and ultimate analyses. It allows for more accurate and realistic modeling of waste-to-energy processes.
- The study applies the model to a specific case study in Mexico City, utilizing monthly waste data to capture seasonal variations. This geographical focus provides valuable insights into the practical implementation of sustainable waste management strategies in Latin America, highlighting the model’s adaptability and relevance to real-world scenarios.
- The proposed model is scalable and adaptable to different regions and contexts. While applied to Mexico City in this study, the methodology can be easily modified to suit other geographical locations, making it a versatile tool for global waste management practices.
- The proposed model offers a decision-making framework that balances economic, environmental, and social objectives. By presenting a Pareto front to illustrate trade-offs and optimal solutions, the study provides a valuable tool for policymakers to formulate sustainable waste management policies on a global scale.

This work aims to propose a decision-making framework for multiple decision-makers. Each of the above objectives can be weighted to determine the optimal solutions. This study analyzes Pareto optimal solutions sets for integrated waste management planning. These solutions provide decision-makers with diverse options, allowing them to select solutions based on specific criteria, priorities, and constraints. By considering various combinations of WtE technologies and evaluating economic, environmental, and social factors, the Pareto optimal solutions sets enable decision-makers to make informed choices in developing sustainable waste management plans.

## Waste to energy technologies targeted

WtE technologies refer to the various methods of generating energy from waste materials. These technologies are designed to convert waste into usable forms of energy, such as electricity or heat, which can then be used for various purposes^34^. Furthermore, Table 1 describes the technologies considered in this work.

**Table 1:** Generalities of WtE technologies.

Technology	Description	Diagram
Incineration (Inc)	Waste materials are burned at high temperatures in a controlled combustion process with excess oxygen. This produces heat energy and transforms organic matter into gases such as carbon dioxide (CO_2_) and water vapor (H_2_O). Non-combustible materials yield a minimal byproduct known as ash. The heat energy obtained can be used to generate steam and electricity, supply electricity for plant consumption, provide hot water for domestic heating, and facilitate other energy retrieval applications.	
Anaerobic digestion (AD)	It is a biological process based on the decomposition of organic matter in the absence of oxygen by using microorganisms to generate biogas, predominantly composed of methane (CH_4_) and carbon dioxide (CO_2_). The syngas obtained through steam methane reforming can be employed in a gas turbine, internal combustion engine, or combined cycle plant to produce electricity and heat. The remaining digested material, digestate, can be used as a nutrient-rich fertilizer.	
Pyrolysis (Py)	It is a thermal decomposition process that transforms organic waste into three main products: solid biochar, liquid bio-oil, and syngas. The process involves heating the material without oxygen, which causes chemical breakdown. The remaining solid residue is a carbon-rich material used primarily for soil amendment, water filtration, and heat or electric power production. The liquid product can be further refined into biofuels, and the gaseous product is useful for heat and electricity generation and serves as a chemical feedstock in various industrial processes.	
Gasification (Gsf)	A thermochemical conversion process subjects the carbon-based feedstock to high temperatures with controlled oxygen or air supply. It leads to partial matter combustion, generating a versatile gas mixture known as syngas, which comprises carbon monoxide (CO), hydrogen (H_2_), carbon dioxide (CO_2_), methane (CH_4_), and other trace gases. This gaseous fuel can be utilized for applications in heat and electricity generation, as well as in liquid fuel and chemical production through downstream industrial processes. .	

Each WtE technology has advantages and disadvantages, and its suitability depends on various factors such as the type, amount, composition, flow, scale of operation, infrastructure, energy requirements, and economic and environmental considerations. Inc., for instance, converts waste into energy, which reduces landfill use, but it also emits harmful gases and can be expensive. On the other hand, AD processes organic waste to produce biogas, which reduces methane emissions and produces fertilizer, but it has specific constraints and setup costs. Py efficiently transforms various wastes into fuels with lower emissions but faces challenges such as feedstock quality. Finally, Gsf generates syngas efficiently, with lower emissions than incineration, but it requires expertise and can be costly. Despite these drawbacks, WtE technologies contribute to a circular economy by reducing landfill reliance and promoting sustainability.

## Description of the problem

**Figure 1:**
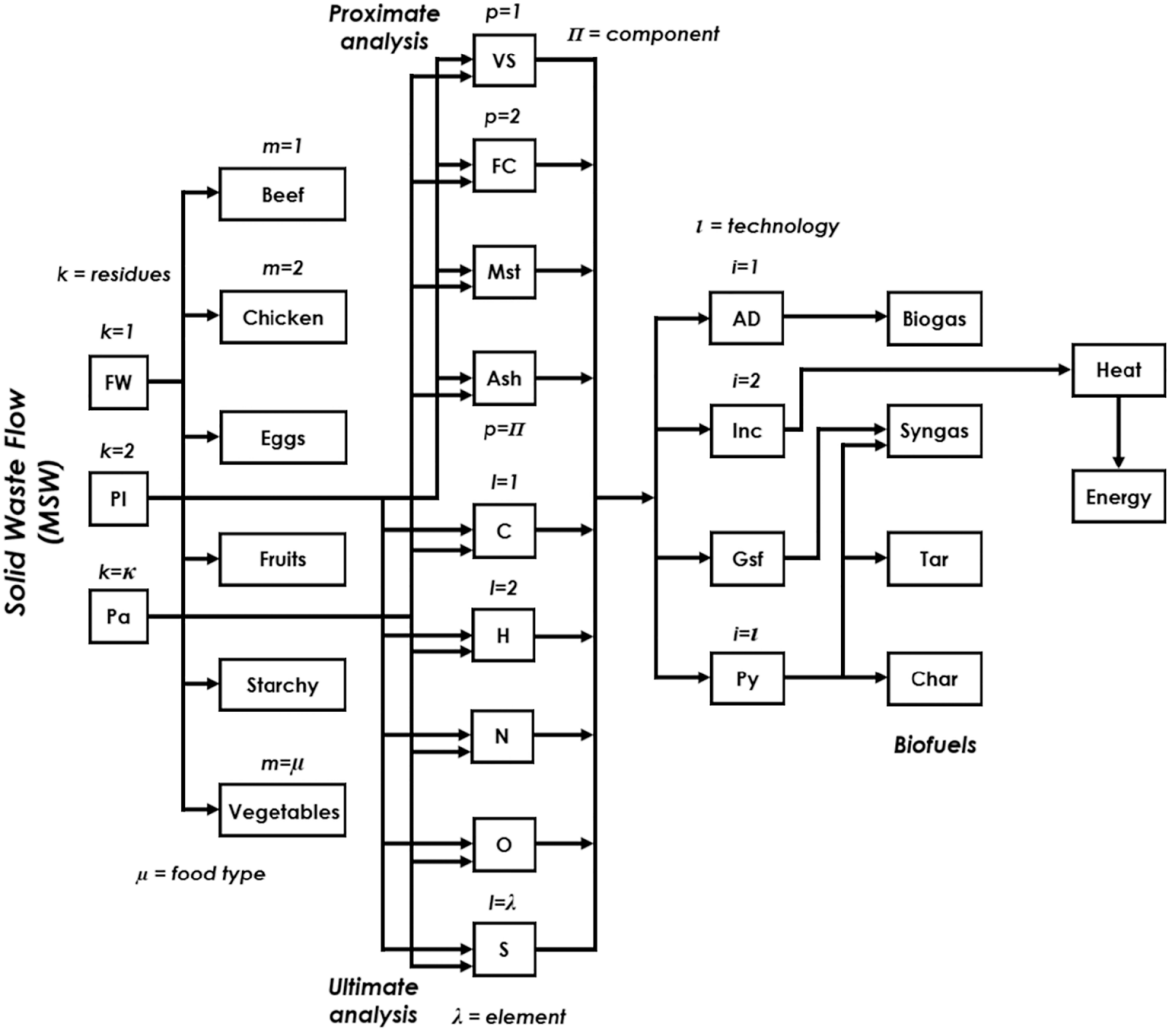
Schematic of the proposed superstructure for waste conversion. This flowchart illustrates the process of converting different types of waste into various forms of energy and biofuel through proximate and ultimate analysis and waste-to-energy conversion technologies.

The problem addressed in this work is schematically described in Fig. 1. Specifically, this study aims to provide an optimal solution to the effective management of MSW by optimizing the selection of resources and technologies. Thus, the problem statement is formulated based on the following inputs:

- *Solid waste* monthly distribution in the selected country as a case study country. This information provides insights into the amount and type of waste generated in each country.
- *Efficiency of the power plants*. The efficiency of the power plant plays a significant role in determining the amount of electricity that can be generated from the waste and, thus, the profitability of the process.
- *Economic factors* Factors such as social discount rate, tipping fee, and labor and land cost should be considered during the optimization process, as they are important in assessing the economic viability of the MSW management process.
- *Electricity prices* Considering the electricity prices in each country affects the profitability of the management process and must be considered during the optimization process.
- *Emission factors by energy generation* The emissions associated with the generation of electricity from different sources vary. Therefore, it is important to consider the grid emissions factors associated with each energy generation method.

This work proposes a superstructure model, presented in Fig. 1, which can aid in developing effective strategies and decision-making by providing a comprehensive representation of MSW composition.

The resources can be processed using different WtE technologies. These technologies produce different fuels used to generate electricity in a power plant. The integrated network is designed to optimize the selection of resources and technologies to replace fossil-based power and fuels. The first step in this process involves determining the MSW composition for the city, country, or region (depending on the case study). Each has a segregation route, which can be further segregated into plastic, paper, and food waste based on the general composition. Food waste is then classified by type, and proximate and ultimate analyses are performed. This information determines the composition per ton of MSW, a parameter independent of the optimization process. Once the composition is known, it is scaled with the mass flow variable to determine the waste flow that enters each WtE technology. Each technology has its specific route for fuel production, and the fuel flow is determined using mathematical models that govern the processes involved. The resulting fuels are then sent to their respective power plants, converted into electricity, and sold to each country’s electricity grid. This approach generates profits and reduces emissions, which are the primary objective functions that need to be optimized.

This study addresses the complex issue of selecting resources and technologies to efficiently manage MSW while considering economic, environmental, and social factors. It is important to note that the operational policy takes a multi-period approach, and the management is subject to a set of nonlinear constraints based on the waste type of each entity, denoted as *j*. Furthermore, the formulation implies the following:

- The mathematical formulation of the model to determine the optimal operation policy with respect to MSW composition.
- The model evaluates the energy generation of four WtE technologies based on their electricity generation potential.
- The economic evaluation of the WtE system is subject to the evaluation of capital costs (Capex), operational costs (Opex), and incomes, considering scale economies and differences between countries.
- Environmental assessment. It involves the estimation of life-cycle greenhouse gas emissions generated by the process and avoided because of the displacement of fossil fuels.

In addition, for the optimization, the following assumptions are made:

- Waste enters each facility ready for immediate conversion. However, the economic models include consolidated pre-treatment costs for each process and technology.
- The composition of waste streams (food waste, plastics, paper) and their elemental makeup are assumed constant.
- Emission factors for GHG emissions from different technologies are fixed.
- Revenue from electricity sales, carbon credits, and gate fees are constant.
- Costs associated with labor and land are based on predefined parameters and remain constant.
- A fixed annualization factor is used to calculate annual costs from capital investments.
- A constant value for the SCC is uniformly applied across all technologies.

The problem is approached as a multi-objective optimization approach aimed at achieving multiple objectives simultaneously. It seeks to maximize economic benefits and maximize avoided greenhouse gas emissions. Here, it is important to that note that maximizing emissions avoidance means minimizing the environmental impact by reducing the amount of greenhouse gases released into the atmosphere. In addition, a social metric is introduced to assess the total social cost of carbon (TSCC). Finally, the thermo-chemical analysis of the processes is utilized to establish an optimal operational policy and system size based on the waste composition in the selected city of a specific country. This analysis allows for selecting appropriate resources and technologies that can effectively substitute fossil-based power and fuels, further contributing to reducing GHGT and the SCC.

## Methods

**Figure 2:**
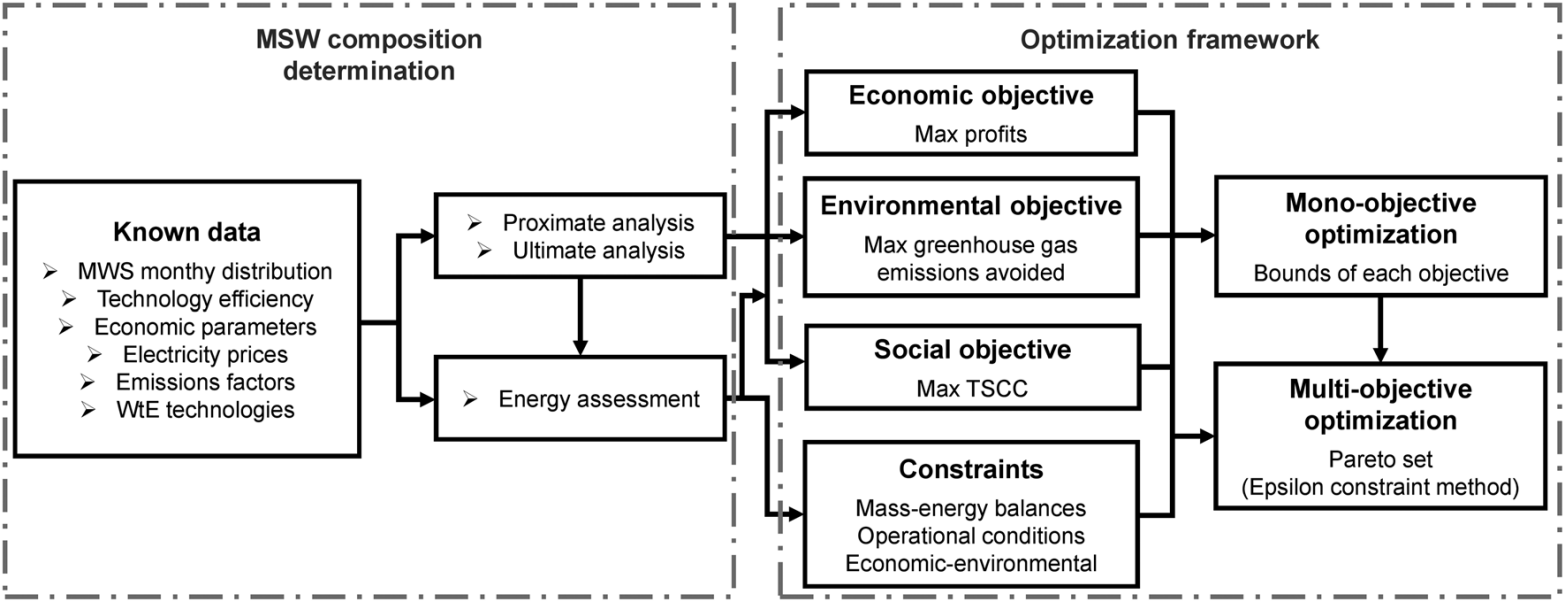
This flowchart outlines the sequential steps for developing waste management solutions. It includes data acquisition and multi-objective optimization, while integrating economic, environmental and social objectives within a structured decision-making framework.

In general, Fig. 2 shows the methodology for developing and testing an optimization model consists of the following steps: The first step is the problem statement, which involves gathering and analyzing relevant information about the problem. For waste management, this includes conducting a comprehensive literature review on WtE technologies, the composition and distribution of MSW, its specifications, costs, environmental impact, and regulatory requirements. The second step is the mathematical formulation, defining the variables, equations, parameters, and objective functions. The third step is multi-objective optimization, where this approach is used to optimize MSW management alternatives by considering both economic profits and environmental benefits. The fourth step is finding optimal solutions. A suitable algorithm for the proposed model is selected to obtain numerical results. Pareto-optimal solutions are also determined, which represent a balance between the different objectives, where improving one objective would entail the deterioration of another. Finally, refinement, validation, and verification are presented in the results and discussion section.

## Optimization formulation

The proposed model is based on the superstructure depicted in Fig. 1, which facilitates the identification of the most effective approach by optimizing the selection and sizing of diverse technologies. It is based on factors such as waste composition, resource availability, energy efficiency, profits, costs, and environmental impact. Equations model the proposed technologies and waste characterization. Moreover, the mass and energy balances, economic and environmental constraints, and various nonlinear constraints that are inherent to the design and operation of the system and decision variables are required.

### Design formulation

The mathematical model is based on the diagram shown in Fig. 1. Moreover, the presented multi-period model considers the following sets:

$$\begin{aligned} t&=\left\{ 1,2,3,...,\tau \right\} \\ j&=\left\{ 1,2,3,...,\chi \right\} \\ i&=\left\{ 1,2,3,...,\iota \right\} \\ k&=\left\{ 1,2,3,...,\kappa \right\} \\ m&=\left\{ 1,2,3,...,\mu \right\} \\ l&=\left\{ 1,2,3,...,\lambda \right\} \\ p&=\left\{ 1,2,3,...,\Pi \right\} \\ \end{aligned}$$

Set $\chi$ represents a group of cities in different countries on which the analysis will focus. In this case, *j*=$\left\{ Entity: City,\ Country,\ Region,\ Institution\right\}$. Moreover, the time resolution is used to determine the monthly operating policy (t), thus, $\tau$=*12*. Besides, the set *i* encompasses all available technologies for waste management *i*=$\left\{ AD,\ Inc,\ Gsf,\ Py\right\}$ Also, food, paper, and plastic waste must be classified and grouped into set *k*, where $\kappa$=3. Additionally, set *m* is used to classify food waste into subcategories such as meat, poultry, eggs, vegetables, and fruits. On the other hand, the set *l* is used for representing each of the elements that are used in the ultimate analysis. In this case, five elements are considered, $\lambda$ = 5. Finally, the set *p* involves all the components in the proximate analysis contained in each residue.

### MSW composition modelling

The MSW generated in a country (*MSWj*) can be calculated by adding up the quantities of different types of waste (${\varphi _k}$) generated during a given period of time (*t*). Here, *k* corresponds to each element of the set $\kappa$ and refers to plastic (${Pl}_{j,t}$), paper (${Pa}_{j,t}$), and food waste (${FW}_{j,t}$) as solid waste, while inert components such as metals and glass are excluded. The equation below summarizes this relationship:

1a $$\begin{aligned} {MSW}_{j,t}=\mathbf {\sum }_{k=1}^{\kappa }{\varphi _{k,j,t}=}\left\{ \varphi _{FW,j,t},\varphi _{Pl,j,t},\varphi _{Pa,j,t} \right\} \ \ \ \ \ \ \ \ \ \ \ \forall j\in \chi \, t\in \tau \ \ \ \ \ \ \ \ \ \ \ \ \ \ \ \end{aligned}$$

Furthermore, the total waste stream can be calculated by summing the total waste streams sent to each waste technology (*i*):

1b $$\begin{aligned} {MSW}_{j,t}=\sum _{i=1}^{\iota }\sum _{k=1}^{\kappa }{\varphi _{k,j,i,t}}\ \ \ \ \ \ \ \ \ \ \ \forall j\in \chi \, t\in \tau \ \ \ \ \ \ \ \ \ \ \ \ \ \ \ \end{aligned}$$

Now, to estimate the quantities of waste $(\varphi _k)$, it is necessary to determine the mass fraction $(\gamma ^{mf})$ of each type of waste and for each country. This fraction varies from country to country; therefore, a fixed value of mass fraction has been used as a parameter. Thus, to determine the quantity of each type of waste generated in a given month $(\varphi _{k,j,t})$, one can simply multiply its mass fraction by the total amount of waste produced during that period.

1c $$\begin{aligned} \varphi _{k,j,t}=\gamma _{k,j}^{mf}\cdot F^{MSW}_{j,t}\ \ \ \ \ \ \ \ \ \ \ \ \ \ \ \ \ \forall j\in \chi \ ,\forall k\in \kappa ,\forall t\in \tau \end{aligned}$$

These equations provide a framework for quantifying the amount of MSW generated and processed in a given country.

#### Ultimate and proximate analysis

The MSW composition modeling can be conducted through and proximate analysis to gain insights into waste makeup in a particular area. The ultimate analysis involves identifying the elements in the waste stream, while proximate analysis determines the amount of moisture, ash, volatile matter, and fixed carbon in the waste. These analyses obtain chemical and physical properties of MSW, which contribute to waste management.

First, the total mass of each residue ($$\varphi _{k,j,t}$$) must correspond to the total sum of its constituent elements ($$n_{l,k})$$, which include carbon (C), hydrogen (H), nitrogen (N), oxygen (O), and sulfur (S), all of which are encompassed by the set $$\lambda$$. The ultimate composition of a particular residue *k* can be expressed mathematically as:

2a $$\begin{aligned} \varphi _{k,j,t}=\sum _{l=1}^{\lambda }{n}_{l,j,k,t}=\left\{ n_{C,k,j,t},n_{H,k,j,t},n_{N,k,j,t},n_{O,k,j,t},n_{S,k,j,t} \right\} \ \ \ \ \ \ \ \ \ \ \ \ \ \ \forall j\in \chi \ ,\forall k\in \kappa ,\forall t\in \tau \end{aligned}$$

where $n_{C},n_{H},n_{N},n_{O},$ and $n_{S}$ are the number of carbon, hydrogen, nitrogen, oxygen, and sulfur atoms, respectively, in the residue *k* for each country *j* in a given period of time *t*.

Similarly, proximate analysis determines the mass of component *p* in residue *k*, which includes volatile solids (VS), fixed carbon (FC), moisture (Mst), and ashes (Ash) contained in each residue.

2b $$\begin{aligned} \varphi _{k,j,t}=\sum _{p=1}^{P}{m}_{p,j,k,t}\ \ \ \ \ \ \ \ \ \ \ \ \ \ \ \ \ \forall j\in \chi \ ,\forall k\in \kappa ,\forall t\in \tau \end{aligned}$$

where, $m_{p,k,j,t}=\left\{ m_{VS,k,j,t},m_{FC,k,j,t},m_{Mst,k,j,t},m_{Ash,k,j,t} \right\}$. In addition, to estimate the composition of a residue, it is necessary to determine the mass fraction of proximate components $(\sigma _{p,k})$ and their proportion in the function of the total mass of residue ($\varphi _{k}$).

2c $$\begin{aligned} \sigma _{p,k,j,t}=\frac{m_{p,j,k,t}}{\varphi _k}x100 \ \ \ \ \ \ \ \ \ \ \ \ \ \ \ \ \ \forall j\in \chi \ ,\forall k\in \kappa ,\forall p\in \Pi , \forall t\in \tau \end{aligned}$$

Now, to classify food waste for a specific country and time period $(\varphi _{FW,j,t})$, it is categorized according to the discarded food types, denoted by $\theta _m$. It can be achieved by calculating the total amount of each type of food wasted, represented by the sum of various food categories such as beef (B), chicken (Ch), eggs (Egg), fruits (Fr), starchy food (Sty), and vegetables (V).

2d $$\begin{aligned} \varphi _{FW,j,t}=\sum _{m=1}^{\mu }\theta _{m,j,t}=\left\{ \theta _{B,j,t},\theta _{Ch,j,t},\theta _{Egg,j,t},\theta _{Fr,j,t},\theta _{Sty,j,t},\theta _{V,j,t} \right\} \ \ \ \ \ \ \ \ \ \ \ \ \ \ \forall j\in \chi \ ,\forall t\in \tau \end{aligned}$$

In the last classification, to estimate the amount of each type of the residue $\theta _{m,j,t}$, it is necessary to determine their mass fraction $(\psi _{m,j,t})$ and their proportion in function of the total mass of residue ($\varphi _{k}$).

2e $$\begin{aligned} \theta _{m,j,t}=\psi _{m,j,t} \cdot \varphi _{FW,j,t} \ \ \ \ \ \ \ \ \ \ \ \ \ \ \forall m\in \mu \ ,\forall j\in \chi ,\forall t\in \tau \end{aligned}$$

It is important to note that mass fractions $\left( \psi _{B,j,t},\psi _{Ch,j,t},\psi _{Egg,j,t},\psi _{Fr,j,t},\psi _{Sty,j,t},\psi _{V,j,t} \right)$ may vary by country and time of year due to variation in the amount of food waste $(\varphi _{FW,j,t})$.

### Waste to energy modeling

Waste energy refers to converting waste heat or other waste energy into useful energy, as Section 2 addressed. It can be accomplished through different WtE technologies $(\iota )$. In this work, four technologies were analyzed: anaerobic digestion, incineration, pyrolysis, and gasification. Each method allows for the efficient conversion of waste materials into energy that can be used to generate electricity. Besides, it is important to note that all calculations were made on a dry volume basis. This subsection examines the application of each WtE technology model:

#### Anaerobic digestion modeling

First, a mass balance for the anaerobic digestion (AD) technology is carried out and relates the input and output flows of each component as follows:

3a $$\begin{aligned} \varphi _{FW,AD,j,t}+Water_{j,t}^{AD}=F_{AD,j,t}^{CH_4} +F_{AD,j,t}^{Dig}\ \ \ \ \ \ \ \ \ \ \ \ \ \ \forall j\in \chi \ ,\forall t\in \tau \end{aligned}$$

where $F_{AD,j,t}^{CH_4}$ and $F_{AD,j,t}^{Dig}$ are the methane and digestate flows rate produced from AD in each country *j*. However, the efficiency and output quality of anaerobic digestion are directly influenced by the total waste flow ($\varphi _{FW, AD,j,t})$ and the quantity of water($Water_{AD,j,t}$) added to the process.

In addition, AD is a process that involves the decomposition of organic waste in the absence of oxygen. This process produces biogas, which can be used to generate electricity $\ \left( E_{AD,j,t}\right)$. The energy obtained from anaerobic digestion comes from two products, methane $\ \left( E_{AD,j,t}^{CH_4}\right)$ and digestate $\ \left( E_{AD,j,t}^{Dig}\right)$. It is important to note that methane contains most of the total energy recoverable from anaerobic digestion. However, this paper considered the digestate for a fair comparison in energy terms.

3b $$\begin{aligned} E_{AD,j,t}=E_{AD,j,t}^{CH_4}+ E_{AD,j,t}^{Dig}\ \ \ \ \ \ \ \ \ \ \ \ \ \ \forall j\in \chi \ ,\forall t\in \tau \end{aligned}$$

However, the production of electrical energy from methane (Eq. 3c) and from digestate (Eq. 3d) depends on the electrical efficiency ($\eta ^{AD}$) of methane and digestate energy as it differs due to their distinct physical states after anaerobic digestion: methane energy is in the form of gas fuel (G), while digestate energy is in the form of solid fuel (S).

3c $$\begin{aligned} E_{AD,j,t}^{CH_4}= & {} LHV^{CH_4}\cdot \eta ^{AD,G}\ F_{AD,j,t}^{CH4}\ \ \ \ \ \ \ \ \ \ \ \ \ \ \forall j\in \chi \,\forall t\in \tau \end{aligned}$$

3d $$\begin{aligned} E_{AD,j,t}^{Dig}= & {} LHV^{Dig}\cdot \eta ^{AD,S}\ F_{AD,j,t}^{Dig}\ \ \ \ \ \ \ \ \ \ \ \ \ \ \forall j\in \chi \,\forall t\in \tau \end{aligned}$$

The flow of methane ($F_{AD,j,t}^{CH4}$) in the AD process is determined by the application of the Boyle equation, which considers the mass balance with other ultimate flows of food waste $(n_{l})$. However, it should be noted that the fixed carbon fraction cannot be converted into gaseous fuel, thus only the volatile solids flow of food waste $(m_{VS,FW,j,t})$ is utilized. In order to achieve a more precise outcome, an adjustment factor $(\delta ^{ADJ})$ is incorporated, following the methodology proposed by^35^.

3e $$\begin{aligned} F_{AD,j,t}^{CH4}=\delta ^{ADJ}\ \left( \frac{n_{C,FW,j,t}}{24}+\frac{n_{H,FW,j,t}}{8}, ...,\frac{n_{S,FW,j,t}}{128}\right) m_{VS,FW,j,t} \cdot \varphi _{FW,AD,j,t}\ \ \ \ \ \ \ \ \ \forall j\in \chi \ ,\forall t\in \tau \end{aligned}$$

#### Incineration modeling

The energy generation from incineration ($E_{Inc,j,t}$) is determined through a statistical regression analysis described by^36^. In this model, the energy estimation is derived exclusively from the flow of the carbon element ($n_{C,Inc,j,t}$) and the moisture flow from MSW $(m_{Mst,Inc,j,t})$, while considering the efficiency of the incineration plant ($\eta ^{Inc}$). The mathematical equation for calculating the energy generation from incineration is as follows:

4 $$\begin{aligned} E_{Inc,j,t}=\eta ^{Inc} \cdot F_{Inc,j,t}^{MSW} \left( 20.97+40{n_{C,Inc,j,t}}^2-5.464 n_{C,Inc,j,t}^{0.5}-2.45 m_{Mst,Inc,j,t}\right) \ \ \ \ \ \ \ \ \ \ \ \ \forall j\in \chi \ ,\forall t\in \tau \end{aligned}$$

#### Pyrolysis modeling

To identify the inputs and outputs of the system, a mass balance into the pyrolysis system is carried out:

5a $$\begin{aligned} F^{MSW}_{py,j,t}=F_{Py,j,t}^{Char}+F_{py,j,t}^{L}+F_{Py,j,t}^{G}\ \ \ \ \ \ \ \ \ \ \ \ \forall j\in \chi \ ,\forall t\in \tau \end{aligned}$$

$F^{MSW}_{py,j,t}$ represents the inlet mass flow rate of the MSW being fed into the pyrolysis system. While the right side of the equation consists of three terms: $F_{Py,j,t}^{Char}$ represents the mass flow rate of solid char, which is the carbonaceous residue produced during pyrolysis, $F_{Py,j,t}^{L}$ represents the mass flow rate of liquid products, such as bio-oils or pyrolysis oils, which are generated during the process, and $F_{Py,j,t}^{G}$ represents the mass flow rate of gaseous products, including syngas, methane, carbon dioxide, and other volatile organic compounds.

In this way, the energy generation from pyrolysis ($E_{Py,j,t}$) is derived from the fuels produced and their respective heating values ($LHV^{Char}$, $LHV^{L}$, $LHV^{H_2}$, $LHV^{CO}$, $LHV^{CH_4}$). The products of pyrolysis can be classified into char flow ($F_{Py,j,t}^{Char}$), pyrolytic liquid flow ($F_{Py,j,t}^{L}$), and gaseous fuels ($F_{Py,j,t}^{\ H_2}$, $F_{Py,j,t}^{\ CO}$, $F_{Pyro,j,t}^{{CH}_4}$). Each fuel type requires a power generation process directly influenced by the efficiency of its respective state of matter ($\eta$).

5b $$\begin{aligned} E_{Py,j,t}&=LHV_{Py,j,t}^{Char}F_{Py,j,t}^{Char}\eta ^{S}+LHV_{Py,j,t}^{L}\ F_{Py,j,t}^{L}\eta ^{L} \nonumber \\&\quad +\left( F_{Py,j,t}^{\ H_2}LHV_{Py,j,t}^{H_2}+F_{Py,j,t}^{\ CO}LHV_{Py,j,t}^{CO}+\ F_{Pyro,j,t}^{{CH}_4}LHV_{Py,j,t}^{CH_4}\right) \eta ^{G} \ \ \ \ \ \ \ \ \ \ \ \ \forall j\in \chi \ ,\forall t\in \tau \end{aligned}$$

The heating values of well-known compounds such as $\hbox {CO}_2$, $\hbox {H}_2$, and $\hbox {CH}_4$ are listed in tables, while for other compounds, estimations are necessary. The heating value of char ($LHV^{Char}$) was calculated using the model proposed by^37^ as shown in Eq. (5c), whereas the heating value of pyrolytic liquid ($LHV^{L}$) was obtained from^38^. This choice was made due to the similarities in biomass feedstock, process conditions, and ultimate content $(\gamma ^{C,H,O})$.

5c $$\begin{aligned} LHV_{Py,j,t}^{Char}=\frac{(33.2\gamma _{j,t}^{C,\ Char}+176.6\gamma _{j,t}^{H,\ Char}-17.78\gamma _{j,t}^{O,\ Char}+0.702)}{3600}\ \ \ \ \ \ \ \ \ \ \ \ \forall j\in \chi \ ,\forall t\in \tau \end{aligned}$$

In Eq. (5b), the char flow $(F_{Py,j,t}^{Char})$ is estimated based on its ultimate composition $(\gamma ^{C,H,O})$ and depends directly on the pyrolysis temperature $(T^{Py})$. The following equations are used:

5d $$\begin{aligned} F_{Py,j,t}^{Char}=\left( 0.106+2.43e^{\left( \frac{-0.66T^{Py}}{100}\right) }\right) \cdot F^{MSW}_{Py,j,t}\ \ \ \ \ \ \ \ \ \ \ \ \forall j\in \chi \ ,\forall t\in \tau \end{aligned}$$

and,

5e $$\begin{aligned} F_{Py,j,t}^{Char}=F_{Py,j,t}^{C}\gamma _{Py,j,t}^{C}+F_{Py,j,t}^{H}\gamma _{Py,j,t}^{H}+F_{Py,j,t}^{O}\gamma _{Py,j,t}^{O}\ \ \ \ \ \ \ \ \ \ \forall j\in \chi \ ,\forall t\in \tau \end{aligned}$$

the ultimate composition of each element present in the char (C, H, and O) is estimated through a generalized equation that depends on the parameters a, b, and c that vary according to each element and represent the influence of different factors as the pyrolysis temperature.

5f $$\begin{aligned} \gamma _{Py,j,t}^{C,H,O}=a\pm be^{\left( \frac{-cT^{Py}}{100}\right) }\ \ \ \ \ \ \ \ \ \ \ \ \forall j\in \chi \ ,\forall t\in \tau \end{aligned}$$

As well, $F_{Py,j,t}^{G}$ is the total pyrolysis gaseous products flow, and it is obtained from the sum of all flows, hydrogen, methane, carbon monoxide, and carbon dioxide.

5g $$\begin{aligned} F_{Py,j,t}^{G}=F_{Py,j,t}^{\ H_2}+F_{Pyro,j,t}^{{CH}_4}+F_{Py,j,t}^{\ CO}+F_{Py,j,t}^{\ CO_2}\ \ \ \ \ \ \ \ \ \ \ \ \forall j\in \chi \ ,\forall t\in \tau \end{aligned}$$

Now, the Song model was used to calculate mass yields of products^39^. In this one, fuel yields depend exclusively on the temperature and biomass composition. Therefore, the next equations are used to estimate the flows of Eq. (5g):

5h $$\begin{aligned} F_{Py,j,t}^{\ H_2}= & {} 1.145\left( 1-e^{\left( -0.11\frac{T^{Py}}{100}\right) }\right) ^{9.384}\cdot F_{Py,j,t}^{G}\ \ \ \ \ \ \ \ \ \ \ \ \forall j\in \chi \,\forall t\in \tau \end{aligned}$$

5i $$\begin{aligned} F_{Pyro,j,t}^{{CH}_4}= & {} \left( 0.146F_{Py,j,t}^{\ CO}-\theta \right) \cdot F_{Py,j,t}^{G}\ \ \ \ \ \ \ \ \ \ \ \ \forall j\in \chi \,\forall t\in \tau \end{aligned}$$

5j $$\begin{aligned} F_{Py,j,t}^{\ CO}= & {} \frac{F_{Py,j,t}^{\ H_2} F_{Py,j,t}^{G}}{\theta +\frac{0.0429}{\left( 1+\frac{T^{Py}}{632}\right) ^{-7.23}}}\ \ \ \ \ \ \ \ \ \ \ \ \forall j\in \chi \,\forall t\in \tau \end{aligned}$$

It is worth mentioning that pyrolysis at 600ºC produces the highest tar yield, which is the desired product. Therefore, this temperature was set for numerical purposes in this work^40^. In this sense, it is necessary to estimate the pyrolytic liquid flow ($F_{Py,j,t}^{L}$). It consists of tar ($F_{Py,j,t}^{Tar}$) and water ($F_{Py,j,t}^{H_2O}$).

5k $$\begin{aligned} F_{Py,j,t}^{L}=F_{Py,j,t}^{Tar}+F_{Py,j,t}^{H_2O}\ \ \ \ \ \ \ \ \ \ \ \ \forall j\in \chi \ ,\forall t\in \tau \end{aligned}$$

Now, an analysis of ultimate composition is required to determine the total flow of the tar:

5l $$\begin{aligned} F_{Py,j,t}^{Tar}=F_{Py,j,t}^{C}+F_{Py,j,t}^{H}+F_{Py,j,t}^{O}\ \ \ \ \ \ \ \ \ \ \ \ \forall j\in \chi \ ,\forall t\in \tau \end{aligned}$$

In the same way, to estimate the flux of the elements O, H, and C present in the tar, the following generalized equation is used:

5m $$\begin{aligned} F_{Py,j,t}^{C,H,O}=n_{Py,j,t}\left( a+\left( \frac{bT^{Py}}{c} \right) \right) \ \ \ \ \ \ \ \ \ \ \ \ \forall j\in \chi \ ,\forall t\in \tau \end{aligned}$$

Parameters a, b, and c vary according to each element and represent the influence of different factors. Moreover, the flows depend on the pyrolysis temperature $(T^{Py})$.

#### Gasification modeling

The next equation is used to estimate the gasification energy $(E_{Gsf,j,t})$, that is determinate by syngas heat power as follows:

6a $$\begin{aligned} E_{Gsf,j,t}=LHV_{Gsf,j,t}^{CH_4}F_{Gsf,j,t}^{CH_4}+LHV_{Gsf,j,t}^{CO}F_{Gsf,j,t}^{CO}+LHV_{Gsf,j,t}^{H_2}F_{Gsf,j,t}^{H_2} \ \ \ \ \ \ \ \ \ \ \ \ \forall j\in \chi \ ,\forall t\in \tau \end{aligned}$$

By summing up the energy contributions from methane, carbon monoxide, and hydrogen in the syngas. The equation considers the mass flow rates of these components $(F_{Gsf}^{CH_4},F_{Gsf}^{CO},F_{Gsf}^{H_2})$ and their respective lower heating values $(LHV^{CH_4},LHV^{CO},LHV^{H_2})$. In simpler terms, it shows how the heat power of the different syngas components determines the energy released during gasification.

6b $$\begin{aligned}{} & {} F_{Gsf,j,t}^{C}\cdot F^{MSW}_{Gsf,j,t}=\frac{12}{16}F_{Gsf,j,t}^{CH_4}+\frac{12}{28}\ F_{Gsf,j,t}^{CO}+\ \frac{12}{48}\ F_{Gsf,j,t}^{CO_2}\ \ \ \ \ \ \ \ \ \ \ \ \forall j\in \chi \,\forall t\in \tau \end{aligned}$$

6c $$\begin{aligned}{} & {} F_{Gsf,j,t}^{H}\cdot F^{MSW}_{Gsf,j,t}=F_{Gsf,j,t}^{H_2}+\frac{2}{18}\ F_{Gsf,j,t}^{H_2O}+\frac{4}{16}F_{Gsf,j,t}^{CH_4}\ \ \ \ \ \ \ \ \ \ \ \ \forall j\in \chi \,\forall t\in \tau \end{aligned}$$

6d $$\begin{aligned}{} & {} F_{Gsf,j,t}^{N} \dot{F}^{MSW}_{Gsf,j,t}+0.767F_{Gsf,j,t}^{air}=F_{Gsf,j,t}^{N_2}\ \ \ \ \ \ \ \ \ \ \ \ \forall j\in \chi \,\forall t\in \tau \end{aligned}$$

6e $$\begin{aligned}{} & {} F_{Gsf,j,t}^{O} \dot{F}^{MSW}_{Gsf,j,t}+0.233F_{Gsf,j,t}^{air}=\frac{16}{28}F_{Gsf,j,t}^{CO}+\frac{32}{48}F_{Gsf,j,t}^{CO_2}+\frac{16}{18}F_i^{H_2O,Gasi}+\frac{32}{64}F_i^{SO_2,Gasi}\ \ \ \ \ \ \ \ \ \ \ \ \forall j\in \chi \,\forall t\in \tau \end{aligned}$$

6f $$\begin{aligned}{} & {} F_{Gsf,j,t}^{S}\cdot F^{MSW}_{Gsf,j,t}=\frac{32}{64}F_i^{SO_2,Gasi}\ \ \ \ \ \ \ \ \ \ \ \ \forall j\in \chi \,\forall t\in \tau \end{aligned}$$

In addition, the Syngas flow consists of fuel compounds as hydrogen $(F_{Gsf,j,t}^{H_2} )$, carbon monoxide $(F_{Gsf,j,t}^{CO})$, and methane $(F_{Gsf,j,t}^{CH_4})$. It also contains non-fuel compounds that act as inert diluent, including carbon dioxide $(F_{Gsf,j,t}^{CO_2})$, steam $(F_{Gsf,j,t}^{H_2O})$, nitrogen $(F_{Gsf,j,t}^{N_2})$, and sulfur dioxide $(F_{Gsf,j,t}^{SO_2})$. Thus,

6g $$\begin{aligned} F_{Gsf,j,t}^{Syn}=F_{Gsf,j,t}^{H_2}+ F_{Gsf,j,t}^{CO}+F_{Gsf,j,t}^{CO_2}+F_{Gsf,j,t}^{H_2O}+F_{Gsf,j,t}^{CH_4}+F_{Gsf,j,t}^{N_2}+F_{Gsf,j,t}^{SO_2}\ \ \ \ \ \ \ \ \ \ \ \ \forall j\in \chi \ ,\forall t\in \tau \end{aligned}$$

At the same time, each reaction relates its equilibrium constant to product flows^41^:

6h $$\begin{aligned} K_{j,t}^{ Boudouard, rxn}= & {} \frac{\left( F_{Gsf,j,t}^{CO}\right) ^2}{F_{Gsf,j,t}^{CO_2}\ F_{Gsf,j,t}^{Syn}}\ \ \ \ \ \ \ \ \ \ \ \ \forall j\in \chi \,\forall t\in \tau \end{aligned}$$

6i $$\begin{aligned} K_{j,t}^{CO,Shift}= & {} \frac{F_{Gsf,j,t}^{CH_4}\cdot F_{Gsf,j,t}^{H_2}}{F_{Gsf,j,t}^{CO}\cdot F_{Gsf,j,t}^{H_2O}}\ \ \ \ \ \ \ \ \ \ \ \ \forall j\in \chi \,\forall t\in \tau \end{aligned}$$

6j $$\begin{aligned} K_{j,t}^{Methanation}= & {} \frac{F_{Gsf,j,t}^{CH_4}\cdot F_{Gsf,j,t}^{Syn}}{(F_{Gsf,j,t}^{H_2})^{2}}\ \ \ \ \ \ \ \ \ \ \ \ \forall j\in \chi \,\forall t\in \tau \end{aligned}$$

where the equilibrium constants *(K)* for boundary (bd) is $K_{j,t}^{bd}=e^{(\frac{Gibbs,bd}{R\ T^{Gsf}})}$, for CO Shift (COs),is $K_{j,t}^{COs}=e^{(\frac{Gibbs,COs}{R\ T^{Gsf}})}$ and for Methanation (mt), is$K_{j,t}^{mt}=e^{(\frac{Gibbs,mt}{R\ T^{Gsf}})}$ are found at a constant temperature $(T^{Gsf})$ and 1 atm of pressure using standard state Gibbs function of change.

Likewise, the heat that is released by the gasification reaction $Q_{j,t}^{Gsf}$ is determined by energy balance, where the enthalpy of inputs $\left( H_I\right)$ is composed of energy from MSW and airflow. While the enthalpy of outputs $\left( H_Ou\right)$ is the energy in syngas products produced.

6k $$\begin{aligned} Q_{j,t}^{Gsf}=H_{j,t}^{Ou}-H_{j,t}^{I}\ \ \ \ \ \ \ \ \ \ \ \ \forall j\in \chi \ ,\forall t\in \tau \end{aligned}$$

where $H_{j,t}^{I}$ and $H_{j,t}^{Ou}$ are calculated as:

6l $$\begin{aligned} H_{j,t}^{I}&=F^{MSW}_{Gsf,j,t}\left( h^{MSW,f}_{j,t}+Cp^{MSW}T_0\right) +F_{Gsf,j,t}^{O}\ Cp^{O_2}\ T_0+F_{Gsf,j,t}^{N_2}\ Cp^{N_2}\ T_0\ \ \ \ \ \ \ \ \ \ \ \ \forall j\in \chi \ ,\forall t\in \tau \end{aligned}$$

6m $$\begin{aligned} H_{j,t}^{Ou}&=F_{Gsf,j,t}^{H_2}+F_{Gsf,j,t}^{CO}\cdot \ominus ^{hf,CO} +F_{Gsf,j,t}^{CO_2}\cdot \ominus ^{hf,CO_2} +F_{Gsf,j,t}^{H_2O}\cdot \ominus ^{hf,H_2O} +F_{Gsf,j,t}^{CH_4}\cdot \ominus ^{hf,CH_4} \nonumber \\&\quad +F_{Gsf,j,t}^{N_2}\cdot \ominus ^{hf,N_2} +F_{Gsf,j,t}^{SO_2}\cdot \ominus ^{hf,SO_2} \ \ \ \ \ \ \ \ \ \ \ \ \forall j\in \chi \ ,\forall t\in \tau \end{aligned}$$

In Eq. (6l), the term $h^{MSW,f}_{j,t}$ that is the enthalpy of formation of MSW was estimated based on^42^, and MSW-specific heat $\left( Cp^{MSW}\right)$ from^43^. While, in Eq. (6m), $\ominus ^{hf}$ corresponds to the enthalpy of formation. Therefore, the next equations are used:

6n $$\begin{aligned} h^{MSW,f}_{j,t}&=2.326\left( -190.3-1407\left( 12\frac{F_{Gsf,j,t}^{H}}{F_{Gsf,j,t}^{C}}\ \right) \right) \ \ \ \ \ \ \ \ \ \ \ \ \forall j\in \chi \ ,\forall t\in \tau \end{aligned}$$

6o $$\begin{aligned} Cp^{MSW}&=0.00486\ T^{Gasi}-0.21293 \end{aligned}$$

#### Social evaluation

The SCC puts the effects of climate change into economic terms to help policymakers and other decision-makers understand the economic impacts of decisions that would increase or decrease emissions. Therefore, the following equations are required:

7a $$\begin{aligned} SCCA_{j,i,t}=CF\cdot {GHGA}_{i,j,t}\ \ \ \ \ \ \ \ \ \ \ \ \ \ \forall i\in \iota \ ,\forall j\in \chi ,\forall t\in \tau \end{aligned}$$

and

7b $$\begin{aligned} SCCG_{j,i,t}=CF\cdot {GHGG}_{i,j,t}\ \ \ \ \ \ \ \ \ \ \ \ \ \ \forall i\in \iota \ ,\forall j\in \chi ,\forall t\in \tau \end{aligned}$$

where SCCA and SCCG correspond to the TSCC for a specific country *j*, technology *i*, and time period *t* by tons of $$\hbox {CO}_2$$ avoided and generated respectively by the use of the proposed technologies. On the other side, CF is a factor used to estimate the SCC in $ per ton of $$\hbox {CO}_2$$ units^44^. Finally, the social metric equation is:

7c $$\begin{aligned} TSCC_{j}=\sum _{i}^{\iota }\sum _{t}^{\tau }(SCCA_{j,i,t}-SCCG_{j,i,t})\ \ \ \ \ \ \ \ \ \ \ \ \ \ \forall j\in \chi \ \end{aligned}$$

In the above equation, $(TSCC_{j})$ is the Total SCC derived for a year of operation.

## Multi-objective optimization approach

In order to determine the optimal operational policy for waste management, a multi-objective approach is adopted that considers economic and environmental aspects. However, some objectives may contradict each other, which poses a challenge in decision-making. On the one hand, maximizing economic benefits may lead to opting for waste incineration, which increases greenhouse gas emissions. Although incineration generates short-term benefits due to energy efficiency, it also presents environmental and social emissions-related challenges. On the other hand, maximizing avoided emissions involves prioritizing more than one WtE technology, which can increase generation costs. As can be seen, the conflicting objectives imply that such a process cannot be operated close to the Utopia region^45^. Since the optimized process cannot be operated around this region, an operating region where the objectives are compensated is selected. This point can be extracted from a Pareto front. Therefore, constructing such a diagram requires a set of objective functions (Eq. 8a). The description of the method is shown below:

8a $$\begin{aligned}{} & {} \Upsilon =\left\{ Profit, GHGT\right\} \end{aligned}$$

8b $$\begin{aligned}{} & {} Maximize \hspace{0.3cm}\{Profit\}\end{aligned}$$

8c $$\begin{aligned}{} & {} s.t \hspace{0.15cm} GHGT\le \varepsilon _{GHGT} \end{aligned}$$

8d $$\begin{aligned}{} & {} \varepsilon _{i} =\Upsilon ^{UB}_i + \frac{\left( \Upsilon ^{UB}_i - \Upsilon ^{LB}_i \right) }{M} \cdot (m-1),\ \hspace{0.3cm}m=\{1,2,...,M\} \end{aligned}$$

Equations (8b)–(8d) represent the formulation of the $\epsilon$-constraint method^46^. In this case, the original problem is converted to a mono-objective problem. That is to say, one of the objectives is selected arbitrarily as an optimization variable *(Profit)*. In contrast, the second objective is treated as a constraint (*GHGT*). This method allows the construction of a Pareto front using the *m* values in a three-dimensional representation of the objective functions.

### Objective functions

#### Economic objective function

The economic viability is essential in optimizing solid waste management through WtE technologies. Evaluating financial profitability, maximizing the economic value of recovered resources, and considering generated revenues and associated costs enables decision-making support regarding these technologies, thus ensuring long-term sustainability. Therefore, the economic assessment was developed by estimating net revenues and total costs. The total cost for each technology, denoted as $Totex_{i,j}$ is comprised of capital cost $\left( Capex_{i,j}\right)$ and operation and maintenance costs $\left( Opex_{i,j}\right)$, thus:

9a $$\begin{aligned} Totex_{i,j}=Capex_{i,j}+Opex_{i,j}\ \ \ \ \ \ \ \ \ \ \ \ \forall j\in \chi \ ,\forall i\in \iota \end{aligned}$$

Where, the $Capex_{i,j}$ depends on equipment cost (*Equip*), capital-labor cost $(Labor^{Capex})$ and land cost (*Land*).

9b $$\begin{aligned} Capex_{i,j}=Equip_{i,j}+Labor_{i,j}^{Capex}+Land_{i,j} \ \ \ \ \ \ \ \ \ \ \ \ \forall j\in \chi \ ,\forall i\in \iota \end{aligned}$$

In addition, Capex and Opex, scale economy models, based on ton MSW for incineration^47^, anaerobic digestion^48^, gasification^49^, and pyrolysis^50^.

In turn, capital-labor cost and land cost were taken as a proportion of equipment cost and were expressed in Table 3 as coefficients $(\alpha _{i,j})$ and $(\beta _{i,j})$ respectively.

9c $$\begin{aligned} Labor_{i,j}^{Capex}&=\ Equip_{i,j} \alpha _{i,j}\ \ \ \ \ \ \ \ \ \ \ \ \forall j\in \chi \ ,\forall i\in \iota \end{aligned}$$

9d $$\begin{aligned} Land_{i,j}&=\ Equip_{i,j}\beta _{i,j}\ \ \ \ \ \ \ \ \ \ \ \ \forall j\in \chi \ ,\forall i\in \iota \end{aligned}$$

To estimate the equipment cost equivalent for each technology, we utilize the following function:

9e $$\begin{aligned} Equip_{i,j}=a \cdot b (\sum _{t}^{\tau }F^{MSW}_{i,j,t})^{kf}\ \ \ \ \ \ \ \ \ \ \ \ \forall j\in \chi \ ,\forall i\in \iota \end{aligned}$$

where $Equip_{i,j}$ represents the equipment cost for each technology for each country. The parameters a and b are specific to each technology and vary accordingly, and are shown in Table 3. The equipment cost is directly influenced by the cumulative amount of solid waste over a specific period. Additionally, the parameter *kf* is the capital cost scaling factor used to determine how the capital cost of the equipment scales with its size, represented by flow throughput. This factor is essential for accurately estimating the capital costs based on the equipment’s capacity.

In this manner, operation and maintenance costs were distributed into labor operation cost $(Labor^{Opex})$ and non-salaries operation and maintenance cost (*NSOpex*). All these costs were brought to present value based on the life cycle of technology *i* and the discount rate for each country $(r_j)$ to equate to capital costs.

9f $$\begin{aligned} Opex_{i,j}=\frac{Labor_{i,j}^{Opex}+{NSOpex}_{i,j}}{\left( 1+r_j\right) ^t}\ \ \ \ \ \ \ \ \ \ \ \ \forall j\in \chi \ ,\forall i\in \iota \end{aligned}$$

Similarly, incomes were allocated to energy sales and a tipping fee (or a gate fee), paid by anyone who disposed of waste in a landfill. And which were determined by their respective unitary prices $\left( {\Omega }_j^{Sells},\ {\Omega }_j^{Tipping Fee}\right)$. Finally, profit $\left( Profit\right)$ are the differences between the net present value of incomes and total costs.

9g $$\begin{aligned} {Incomes}_{i,j,t}=\frac{E_{i,j,t} {\Omega }_j^{Sells}+ {\Omega }_j^{Tipping Fee}F_{i,j,t}^{MSW}\ }{\left( 1+r_j\right) ^t}\ \ \ \ \ \ \ \ \ \ \ \ \forall j\in \chi \ ,\forall i\in \iota ,\forall t\in \tau \end{aligned}$$

Therefore, the economic objective function is the maximization of the profit of each country (*Profit*) resulting from the incomes (*Income*) after total costs (*Totex*):

9h $$\begin{aligned} {Profit}_{j}=\sum _{i}^{\iota }\sum _{t}^{\tau }({Income}_{i,j,t}-Totex_{i,j})\ \ \ \ \ \ \ \ \ \ \ \ \forall j\in \chi \ \end{aligned}$$

#### Environmental objective function

In addition to economic analysis, an environmental evaluation is carried out through the emissions avoided $\left( {GHGA}_{i,j,t}\right)$, due to the prevention of grid emission factors in each country $\left( {f_{avoid}}_j\right)$, and the emissions generated $\left( {GHGG}_{i,j,t}\right)$ by the operation of the WtE technologies^51,52^, expressed as a factor of emissions generated by each technology $\left( {f_{generated}}_i\right)$.

10a $$\begin{aligned} {GHGA}_{i,j,t}&={f_{avoid}}_i\cdot E_{i,j,t}\ \ \ \ \ \ \ \ \ \ \ \ \ \ \forall i\in \iota \ ,\forall j\in \chi ,\forall t\in \tau \end{aligned}$$

10b $$\begin{aligned} {GHGG}_{i,j,t}&={f_{generated}}_j\ \cdot E_{i,j,t} \ \ \ \ \ \ \ \ \ \ \ \ \ \ \forall i\in \iota \ ,\forall j\in \chi ,\forall t\in \tau \end{aligned}$$

Therefore, the environmental objective function is the total emissions $\left( {GHGT}_{j}\right)$, which consist of the difference between avoided and generated emissions:

10c $$\begin{aligned} {GHGT}_{j}=\sum _{i}^{\iota }\sum _{t}^{\tau }({GHGA}_{i,j,t}-{GHGG}_{i,j,t}) \ \ \ \ \ \ \ \ \ \ \ \ \ \ \forall j\in \chi \ \end{aligned}$$

Thus, by reducing emissions, properly managing hazardous waste, promoting resource conservation, and minimizing reliance on landfills, we can achieve a more sustainable and environmentally friendly approach to waste management through WtE technologies.

### Trade-off solution

Derived from this, the boundaries (lower bound and upper bound) of each objective function are determinate. With this, the utopia and nadir points (UP and NP) are defined.

11a $$\begin{aligned} UP&= \left\{ Profit^{UB}, GHGT^{UB}\right\} \end{aligned}$$

11b $$\begin{aligned} NP&= \left\{ Profit^{LB}, GHGT^{LB}\right\} \end{aligned}$$

It is important to note that the UP represents an infeasible solution defined by the target values (Eq. 11a), and the NP represents the undesirable effects (Eq. 11b) on the objective functions. In addition, the coordinates of the utopia and nadir points are used to scale the objective functions and find a trade-off solution (CS). This solution is defined as one that achieves an adequate balance between all objectives and consists of finding the solution closest to the UP. Therefore, using the variable $\widehat{\Upsilon }$ to scale the objectives according to the next expression:

11c $$\begin{aligned} \widehat{\Upsilon }_i&= \frac{\Upsilon _i - \Upsilon ^{LB}_i}{\Upsilon ^{UB}_i - \Upsilon ^{LB}_i}\nonumber \\ s.t\hspace{0.15cm} 0&\le \widehat{\Upsilon }\le \ 1 \end{aligned}$$

On the other side, the level of preference about an objective function is defined by a weight coefficient ($\omega , 0\leqslant \omega _{i}\leqslant 1$). This leads to obtaining a criteria solution for each stakeholder (Cr).

11d $$\begin{aligned} Maximize\hspace{0.3cm}Cr&= \sum _{i=1}^{I}{\omega _{i}{\Upsilon }_i}. \end{aligned}$$

11e $$\begin{aligned} \sum _{i=1}^{I}\omega _{i}&=1 \end{aligned}$$

the index *i= 1, 2 ...I* denotes the number of objective functions, and $\omega$ is the weight associated with the level of preference over the objective function.

## Case study

**Figure 3:**
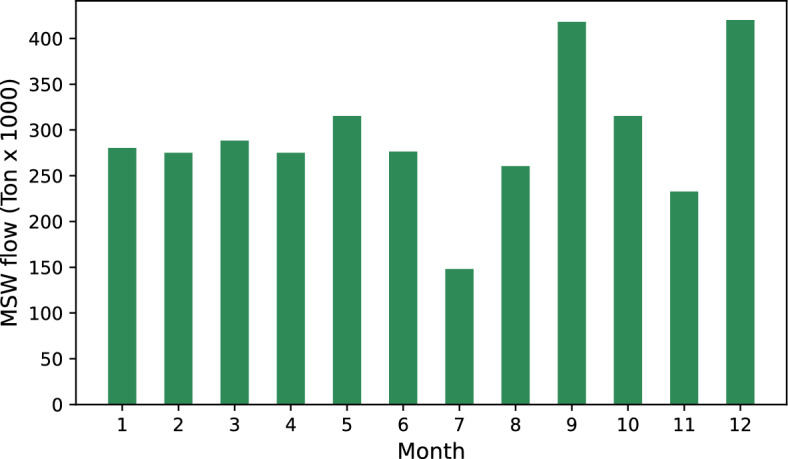
Monthly distribution of MSW flow, measured in thousands of tons, over one year. The vertical bars represent the quantity of waste collected each month, while the dashed blue line indicates the trend over the year.

The selection of Mexico City for this study is crucial due to its dense population and significant waste management challenges, reflecting broader issues in Latin America. The city generates an average of one kilogram of municipal solid waste per person daily, highlighting the urgent need for effective waste management policies. The lack of comprehensive strategies, economic barriers, and cultural practices hinder the adoption of sustainable disposal methods. This study underscores the importance of exploring waste-to-energy (WtE) technologies to mitigate environmental impacts and promote a circular economy, offering a potential model for sustainable waste management in other regions of Latin America. Technical aspects of the optimization process, including the technologies and parameters used, are presented in Table 2. While Table 3 provides additional economic data essential for the model. Moreover, Fig. 3 shows the seasonal solid waste monthly distribution for Mexico City, where waste generation peaks during festive periods in the country, specifically in May, September, November, and December. It is important to mention that data on monthly waste generation in Mexico City are derived from Mexican government reports and are used as representative indicators to assess the challenges associated with waste management in the city. It reflects the total average volume of waste generated by the population.

**Table 2:** Parameters used for optimization.

Concept	Value
Food waste mass fraction	0.68
Plastic mass fraction	0.179
Paper mass fraction	0.141
Power plants efficiency	0.3,0.2,0.4
Pyrolysis temperature ($$^{\circ }\hbox {C}$$)	600
Gasification temperature ($$^{\circ }\hbox {C}$$)	900
Emissions factor avoided (Ton CO_2_ eq./MWh)	0.43
Emissions avoided factor *j* (Ton CO_2_ eq./MWh)	0.05, 0.17, 0.104, 0.15
Life time (years)	20
Energy sell ($/MWh)	0.07
Tipping fee ($/Ton MSW)^53^	0.0106
Carbon cost ($/Ton CO_2_ eq.)^44^	3.50

**Table 3:** Technology and labor costs by plant size.

	CAPEX [Million US$]			OPEX [Million US$/year]			Reference
	Capex (without labor cost)	Capex labor cost proportion (%)	Total capex	Opex (without labor cost)	Opex labor cost proportion (%)	Total opex	
Inc	71.29	16.00	84.87	5.27	23.00	6.80	^54^
AD	26.16	25.00	34.88	4.50	30.00	6.43	
Gsf	122.07	6.70	130.78	5.30	16.00	6.31	^54^
Py	241.24	5.20	254.50	10.83	16.00	12.57	^55^

## Results and discussion

**Figure 4:**
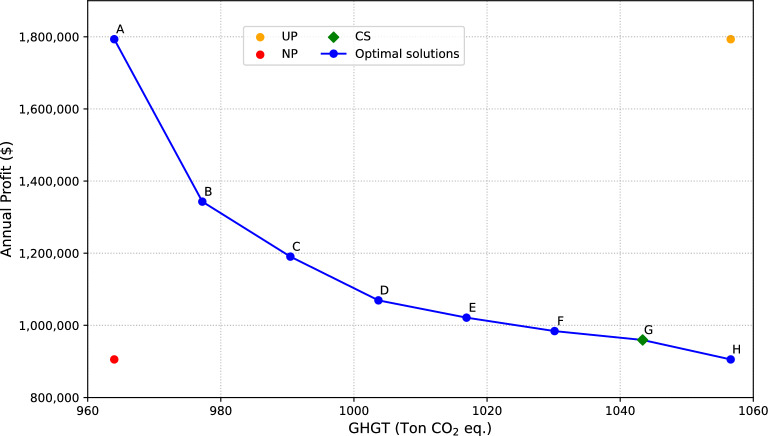
The Pareto front is drawn to find trade-off solutions between annual profit and greenhouse gas emissions. Solution A is the best economic solution, while H is the best environmental solution. The Utopia Point (UP) represents the best theoretical outcome for all objectives, while the Nadir Point (NP) represents the least favorable outcome. The Compromise Solution (CS) denotes a balanced solution.

The mathematical model was implemented using the General Algebraic Modeling System (GAMS) software^56^. It consists of 2815 equations, 2852 variables, and 304 non-linear terms, and the solution for each point of the Pareto curve was solved in an average of 2280 s of CPU time computer with an Intel® $\hbox {Core}^{\textrm{TM}}$ i7 processor at 2.50 Hz with 8 GB of RAM. Due to the multi-linearities involved in the modeling, which leads to non-convex conditions, the NLP model was solved using the global solver BARON^57^. The multi-objective problem is addressed through an epsilon-constraint method to delineate the Pareto front (Fig. 4), where each point represents a trade-off between conflicting objectives based on individual preferences. This approach facilitates the exploration of various scenarios to identify the optimal balance between competing objectives.

The results are presented in three main cases. The first case examines the optimal solutions presented in the Pareto front, analyzing the trade-offs between environmental and economic objectives. The second case involves a detailed comparison between both objectives, exploring their social repercussions. Finally, in the third case, a balanced solution was selected to present the variation to reduce the imbalance between both optimal solutions, considering the criteria that all the objectives have the same importance in the decision-making framework (according to Eq. (11d)). These approaches provide a comprehensive understanding of the implications and trade-offs between the different goals to be considered in the effective management of MSW.

### Case 1: Set of individual optimal solutions

The first case presents all the optimal solutions drawn in the Pareto front (See Fig. 4). Analyzing the trade-offs between environmental and economic objectives aims to identify the best balance that minimizes environmental impact while maximizing economic gains. This analysis sheds light on the most promising strategies for achieving sustainable waste management.

The results demonstrate the influence of dual objectives and how they modify waste management based on specific criteria. These solutions are crucial for strategic economic and environmental waste management decision-making. Each solution represents a set of decisions aimed at optimizing the maximization of economic benefit and minimization of GHGT linked to carbon cost. It is important to highlight the conflict between the maximum benefit and minimum value of the environmental function, i.e., the one emitting the least $\hbox {CO}_2$ into the atmosphere. Addressing this issue entails reducing the annual profit to present more sustainable solutions.

Thus, solution A, highlighted in Pareto front in Fig. 4 as the optimal economic solution, is linked to the highest total profit of $1,793,252.26. This choice is backed by the installation of Incineration technology, yielding substantial annual returns due to its high calorific capacity and production of marketable energy. However, this solution carries the most significant $$\hbox {CO}_2$$ emissions, resulting in the worst environmental scenario with 953 tons of $$\hbox {CO}_2$$ eq. and a high TSCC of $26,261.

Solution H, with a total profit value of $905,566.90, represents 50.4% of the annual profit relative to the optimal economic solution. It reflects substantial variability in profit generation among optimal solutions. Regarding GHGT, Solution H represents the optimal environmental solution by presenting the highest amount of emissions avoided, at 1056.60 tons of $$\hbox {CO}_2$$ eq., 10.8% more than Solution A. This solution combines Gasification and Incineration for waste management, maximizing emissions avoided. The selection is driven by the need to process waste and minimize emissions. Consequently, this solution has a lower impact on the TSCC with $14,400, reducing TSCC impact by 45.1% compared to the economic optimal solution. In this way, intermediate solutions (B, C, D, E, and F) balance economic benefit and environmental responsibility. Different solutions adopt diverse mixes to address economic and environmental challenges in terms of installed plant technology combinations. For instance, Solutions C and D employ Incineration and Anaerobic Digestion technologies, while Solution F incorporates a more diverse set of technologies (Inc, Gsf, and AD). The selection of these technologies is influenced by factors such as the efficiency of energy generation, emissions reduction capabilities, waste processing capacity, and economic viability. The aim is to achieve a combination that aligns with profit maximization and emissions reduction outcomes.

In general, optimal solutions B, C, D, E, F, and G represent net benefits ranging from 54.3 to 69.4% relative to optimal solution A. Regarding the optimal environmental solution (point H), these solutions range between 87.8%. Finally, it is important to note that this analysis provides invaluable insights for decisions in sustainable resource management and climate change mitigation. Each solution represents compromises and trade-offs that must be evaluated considering priorities and specific objectives. The final choice will depend on the weighting and prioritization of factors based on contextual values and goals. In contrast to^58^ study, which identified anaerobic digestion as the optimal technology with 83.85% emission reduction and a cost of energy at 0.0581/kWh, our approach considers a broader spectrum of technologies, achieving a notable emissions reduction of 54.3% to 69.4% across solutions B to G relative to our optimal economic solution (Solution A). Moreover^59^ supply chain optimization in MSW management achieved a 21.6% cost reduction and 28.4% emissions reduction. In this work, Solution H, identified as the optimal environmental solution, achieves a 50.4% profit relative to Solution A while reducing emissions by 10.8%. In addition, the proposed methodology complements mathematical model for MSW circular economy by presenting a multi-objective, multi-period optimization model applied to MSW. As a case study, Mexico City showcases the utility of balancing environmental sustainability and economic objectives. While^60^ achieved significant daily cost reductions of USD 365,000 and avoided 186.43 tons of carbon emissions per day, this work provides a broader perspective by considering various technologies and their combinations, revealing nuanced trade-offs between economic gains and emissions reductions in different scenarios.

### Case 2: Comparing environmental and economic objectives an their social repercussions

**Figure 5:**
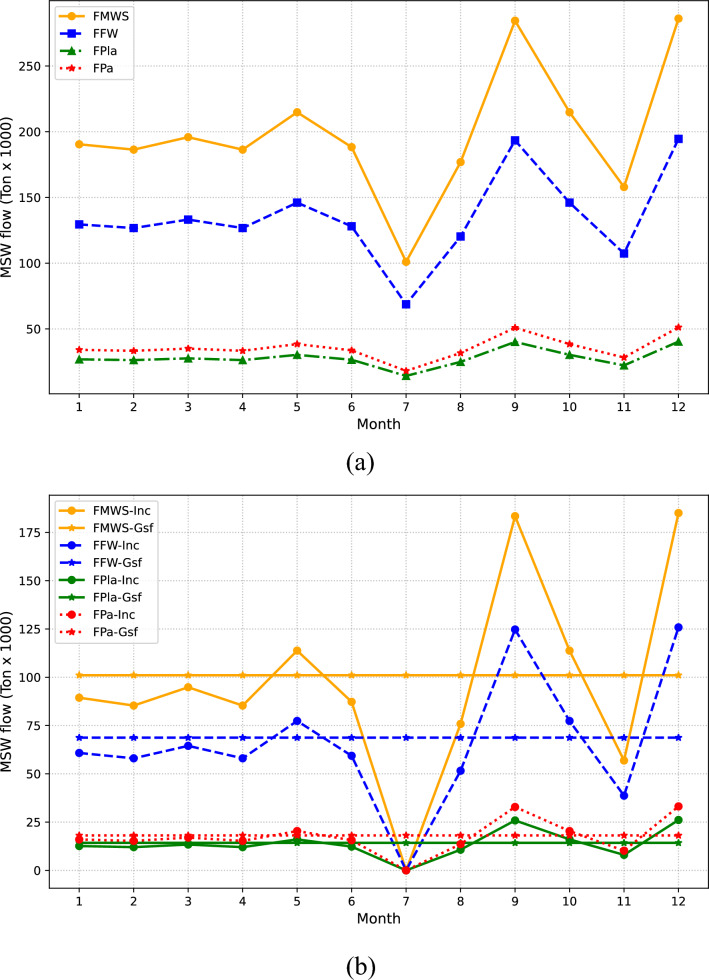
(**a**) Delineates the flow of MSW optimized for economic efficiency, whereas (**b**) reflects an optimization geared towards environmental sustainability. Each line details the progression of food (FW), paper (Pa), and plastic (Pla) waste flow, highlighting the volume managed by the selected technologies every month.

The second case involves a comprehensive comparison between the environmental and economic objectives, focusing on the social implications of different waste management strategies. By considering the social impacts of each approach, we gain valuable insights into the feasibility and desirability of implementing these strategies. Additionally, monthly waste distribution for both the optimal economic solution (Fig. 5a) and the optimal environmental solution (Fig. 5b) is presented in Fig. 5. The figure depicts the flows of three different types of waste: food (FW), paper (Pa), and plastic (Pla). Each flow is shown as a line that represents its quantity throughout the months. The monthly distribution of waste is determined by the specific fractions of each type of waste, considering both the waste type and its inherent quantity in the context of Mexico City. Thus, each waste fraction of FW, Pa, and Pla in each monthly interval reflects the specific proportion of that waste type during that specific period of time. These values align with the data provided in Fig. 3.

Furthermore, variations in the flows of different waste types across the months are observed, establishing inherent connections between waste generation patterns and the inherent fractions of each category. For instance, months with higher waste generation coincide with festive periods in the country, escalating waste production. Specifically, months such as May, September, and December exhibit an average of 135,000 tons of waste per month, comprising 176,000 tons/month of food waste, 35,000 tons/month of paper waste, and 28,000 tons/month of plastic waste. It is clear that in the optimal economic solution, all waste is treated through incineration (Inc) technology. Conversely, in the optimal solution from an environmental standpoint, waste undergoes incineration and gasification (Gsf) processes. Moreover, the distribution presented in Fig. 5b maintains a continuous flow of waste throughout the year (100,000 tons of waste processed monthly), reducing the waste load destined for Inc. This characteristic stems from the objective function aimed at maximizing avoided emissions, thus assigning a significant role to utilizing Gsf. While, Gsf requires less than 50% of the proportional share held by Inc technology.

**Figure 6:**
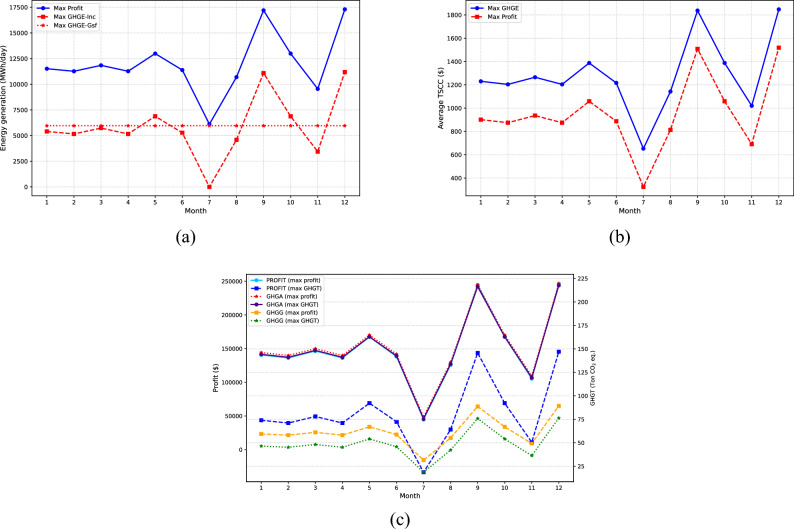
Comparative analysis of economic and environmental waste management strategies. (**a**) Illustrates the energy generated, (**b**) details the societal cost implications, and (**c**) presents the economic-environmental solutions, focusing on maximizing profit and minimizing emissions.

Besides, Fig. 6 provides a comparative analysis of the monthly operational policy for waste management across both optimal solutions. The energy generation is portrayed in Fig. 6a, the TSCC impact in Fig. 6b, and lastly, Fig. 6c presents a comparison between profit and both avoided and generated emissions monthly. Regarding energy generation, it is discernible that the highest output aligns with waste generation profiles. In this context, the peak energy generation occurs during the months of March, September, and December, with an average value of 15,460 MWh/day for the optimal economic solution. It is accompanied by a TSCC of $\$1,366$ and a yield of $\$220,000$. On the other side, within the optimal environmental solution, the average energy generation during the same months is 9500 MWh/day and 5960 MWh/day for incineration and gasification, respectively, coupled with an average TSCC of $\$1716$ and a yield of $\$120,000$. Concerning energy generation, the environmental solution presents a 40% contribution of gasification compared to incineration, as observed in the economic solution. It represents a reduction of 54% in comparison to the economic solution. When it comes to TSCC, the environmental solution has shown significantly higher values. This is mainly because the avoided emissions were much greater, which has increased the TSCC associated with the funds saved as a result of emissions mitigation.

### Case 3: Proposed solution under balanced assumption

The multi-objective optimization analysis yielded important insig hts into selecting the optimal solution for MSW management. In this case, assuming that both objective functions have equal weight in decision-making results in solution C of the Pareto front (see Fig. 4). The methodology aims to balance the two competing objectives while considering the practical constraints and complexities of waste management decision-making. The results of this case highlight a comprehensive approach that meets environmental and economic goals.

**Figure 7:**
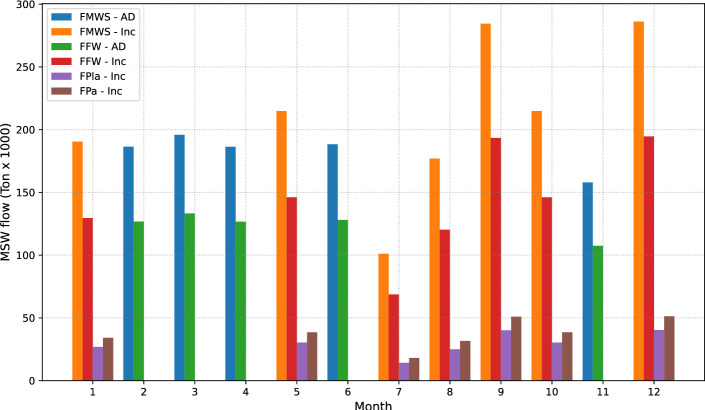
This figure shows the monthly waste treatment plan, which balances economic and environmental goals. The high waste output months use incineration for optimal processing efficiency, while anaerobic digestion is employed during periods of reduced waste generation to enhance environmental objectives.

Figure 7 illustrates the characteristic waste distribution of the attained balanced solution. This solution combines two specific technologies: incineration (Inc) and anaerobic digestion (AD). In this context, it is evident that to achieve the objective of reconciling economic and environmental goals. The operational strategy determines that incineration emerges as the most suitable alternative for processing during months of higher waste generation, such as January, May, September, and December. However, anaerobic digestion is required to attain environmental balance during periods of lower waste production, like February, March, and April. It is worth mentioning that the selection of the appropriate technology in each case is grounded in a detailed analysis of environmental impact, total investment and operational costs, the processing capacity intrinsic to each technology, and its social repercussions. Thus, this balanced solution is established as a strategy that, apart from optimizing financial resources, addresses the demands of environmental sustainability, thus promoting a comprehensive approach backed by robust criteria for design and operation.

**Figure 8:**
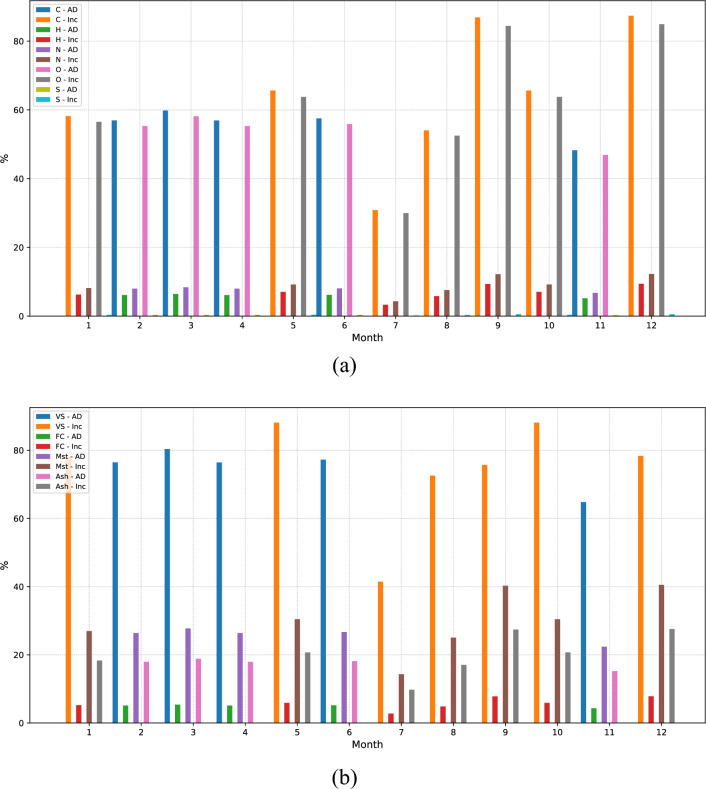
The ultimate and proximate analyses of MSW for the balanced solution are shown in (**a**,**b**). The ultimate analysis reveals the energy content in the waste, while the proximate analysis provides insights into its combustibility. These insights underpin the strategic use of the technologies, optimizing for energy recovery and treatment efficacy.

On the other hand, the proximate and ultimate analyses with respect to this solution are presented in Fig. 8a,b, respectively, as depicted in Fig. 8. In this context, a comprehensive evaluation of solid waste composition is presented, analyzing each mentioned component for a specific quantity of waste per month. From these data, the suitability of different technologies is established based on the types of waste subjected to processing. It is essential for maximizing waste management efficiency, minimizing environmental impact, and making the most of the resources contained within solid waste.

The composition of solid waste is a critical factor in their efficient management, evaluated through proximate and ultimate analyses. These analyses provide essential information about the chemical and physical composition of waste, thereby allowing the determination of the potential to transform the organic matter present in the waste. The proximate analysis presents key components such as volatile solids, carbon, ash, and moisture. These elements are fundamental in understanding the proportion of organic matter that can be degraded through biological or thermal processes. Furthermore, the ultimate analysis, encompassing the determination of carbon (C), hydrogen (H), nitrogen (N), oxygen (O), and sulfur (S) contents, offers a more comprehensive view of the chemical composition of waste. The importance of these analyses is their ability to assist in decision-making when selecting the best technologies for solid waste treatment. By analyzing the values obtained, it is possible to determine which technologies are most effective in processing waste and how much organic matter can be transformed into useful products, such as energy or compost.

**Figure 9:**
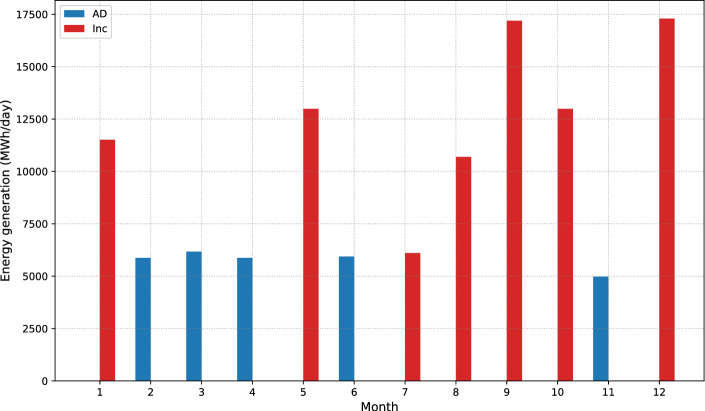
Energy production in balanced solution, where incineration is favored during high-waste months for its higher energy generation than anaerobic digestion. The trend suggests intentionally using incineration to manage larger waste volumes and enhance energy recovery.

Now, it becomes possible to measure the amount of energy produced through waste management using each technology, as shown in Fig. 9. As previously mentioned, this solution involves simultaneous incineration and anaerobic digestion. It is important to highlight that energy production is closely tied to the amount of waste processed; in simpler terms, a larger volume of waste results in a higher energy output. The peak of energy generation is observed during May, September, and December. In these months, energy outputs of 12,987 MWh/day, 17,196 MWh/day, and 17,294 MWh/day, respectively, are achieved through incineration. This notable increase in energy production corresponds to periods of heightened waste accumulation. WHile the anaerobic digestion process predominantly contributes to energy generation during February, March, April, June, and November. In this case, these months demonstrate an average energy generation of 5,767 MWh/day. This specific technology appears to be more effective at converting waste into energy during these specific months, likely due to factors such as the composition of the waste and prevailing environmental conditions. In conclusion, combining incineration and anaerobic digestion techniques represents a versatile waste management approach. It demonstrates how distinct energy outputs react to varying waste quantities and the temporal patterns of waste generation throughout the year.

Table 4 shows monthly energy generation from anaerobic digestion (AD), including produced digestate quantity, energy generated from digestate, and energy generated from methane content in biogas. A significant variation in energy generation is observed in AD during specific months, such as February, March, April, June, and November. On the other hand, the absence of energy generation values in particular months like January, May, and July, among others, suggests the utilization of incineration as a waste treatment method. For example, in March, the month with the highest generation, 185,240 MWh of energy was generated, with 86,830 MWh from digestate and 98,410 MWh from biogas methane. In contrast, in November, the month with the lowest generation, the efficiency of the anaerobic digestion process became evident as it produced 70,020 MWh of energy from 50,590 tons of digestate, despite it being the month with the least energy generated.

**Table 4:** Energy generation capacity from AD (MWh x 1000).

Month	$F^{Dig}$ (Ton x 1000)	$E^{Dig}$	$E^{CH_4}$
2	59.61	82.63	93.65
3	62.62	86.83	98.41
4	59.58	82.58	93.60
6	60.22	83.48	94.62
11	50.59	70.02	79.36

**Figure 10:**
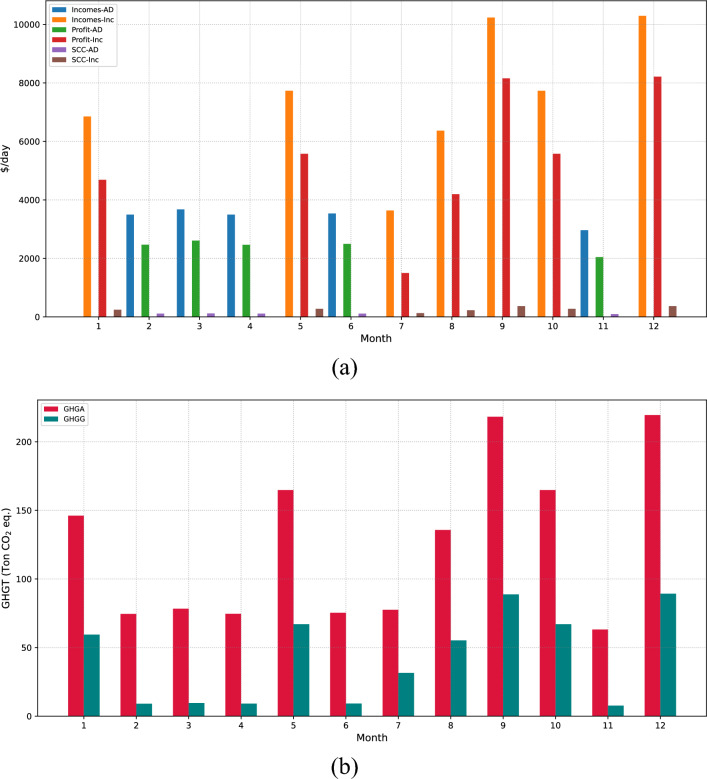
The economic performance of the balaced waste management strategy is presented in (**a**), highlighting financial metrics such as net benefits and revenues. (**b**) Examines the environmental aspect, charting monthly emissions data in relation to the implemented technologies.

Finally, Fig. 10 presents the economic and environmental evaluation of the proposed solution. The economic performance indicators, including net benefit, revenue generated, and social benefits, are shown in Fig. 10a for specific time intervals depending on the technology implemented. In addition, Fig. 10b visually depicts the emissions generated and avoided for each month correlated with the technologies adopted. The profiles are linked to the amount of processed waste managed monthly and correlated with energy generation, as shown in Fig. 9.

## Conclusions

This study presents an innovative multi-objective nonlinear programming (NLP) optimization model to determine the optimal route for converting Municipal Solid Waste (MSW) into energy in a specific city chosen as a case study. In summary, the developed methodology employs a mathematical approach and an epsilon-constrained technique, yielding the Pareto front as a direct result (see Fig. 4). Each point represents an operational policy that balances diverse waste processing technologies to achieve environmental, economic, and social goals. It underscores the value of the proposed methodology as a decision-making tool for policymakers, enabling them to prioritize objectives and formulate sustainable waste management policies globally. Thus, this work provides a systematic approach for addressing complex trade-offs and guiding informed decision-making in waste management. The eight optimal solutions for MSW management, labeled A to H, illustrate the inherent trade-offs that exist between economic and environmental objectives. Solution A, which prioritizes profit through incineration, generates 1.79M$ in profit but emits 954 tons of $$\hbox {CO}_2$$ and incurs a total social cost of carbon of $26,261. In contrast, Solution H, identified as the optimal environmental solution, generates 0.91M$ (50.4% of Solution A) while reducing emissions by 10.8%. Intermediate solutions (B–F) achieve a balance, presenting net benefits ranging from 54.3 to 69.4% relative to Solution A. Highlighting the importance of the trade-offs solutions, Solution H outperforms all others, achieving benefits up to 87.8% in sustainable waste management policies. Besides, the study also found that incineration is the most economically favorable option due to its calorific potential and saleable heat and energy production despite increasing social implications. Alternatively, anaerobic digestion is the most environmentally friendly option, although it may impact economic benefits.

It is important to note that to evaluate the waste management challenges in Mexico City, data on monthly waste production, as reported by the Mexican government, is used as a representative indicator. These statistics demonstrate the total amount of waste generated by the population and provide a solid foundation for demonstrating the practical applicability of the proposed model and its potential to impact operational and waste management policies. The proposed model is general and can be modified to include other technologies, costs, and elements, making it suitable for use in other countries, regions, or institutions. However, it is important to consider specific specifications similar to those outlined in this study when applying the model elsewhere.

For future work, a detailed exploration of the waste management process is crucial, including specific data on population demographics to refine cost-benefit assessments and enhance the accuracy of the model. Additionally, assessments of specific plastic content in the waste stream and comparisons of the environmental performance of WtE technologies with renewable energy sources will be crucial to evaluate long-term sustainability. Finally, incorporating detailed designs of technologies will ensure the technologies are suitable for the waste flow characteristics.

## Data Availability

All data generated, and all parameters used in this study are reported and citepd within the manuscript.

## Acronyms

MSW: Municipal Solid Waste
NLP: Non-linear programming
LATAM: Latin America
WtE-AD: Anaerobic energy
WtE: Waste-to-energy
Pl: Plastic
Pa: Paper
AD: Anaerobic Digestion
Inc: Incineration
Py: Pyrolysis
Gsf: Gasification
FW: Food Waste
VS: Volatile Solids
Ash: Ash
Mst: Moisture
FC: Fixed carbon
G: Gas
L: Liquid
UB: Upper Bound
LB: Lower bound
UP: Utopia point
NP: Nadir point

## Variables

*Totex*: Total cost ($)
*CS*: Trade-off solution
$$\widehat{\Upsilon }_2$$: Normalized environmental function
$$\widehat{\Upsilon }_1$$: Normalized economic function
*Incomes*: Incomes net present value ($)
*Profit*: Profit net present value ($)
*SCC*: Social Carbon Cost ($/Ton)
*Opex*: Operational costs ($)
*Capex*: Capital costs ($)
$$\varphi$$: Amount of waste (Ton)
*n*: Number of atoms of each element *l*
*m*: Mass of component *p* in residue *k*
*Water*: Amount of water (Ton)
*E*: Energy (MWh)
*F*: Flows (Ton)
*LHV*: Low Heating Value
*K*: Equilibrium constant
*GHGT*: Total Greenhouse Gas Emissions ($$\hbox {kg CO}_2$$ eq.)
*GHGA*: Greenhouse Gas Emissions Avoided ($$\hbox {kg CO}_2$$ eq.)
*GHGG*: Greenhouse Gas Emissions Generated ($$\hbox {kg CO}_2$$ eq.)
*Syn*: Syngas flow (Ton)

## Parameters

$$\gamma ^{mf}$$: Mass fraction of each type of waste
CF: Carbon cost factor ($/MWh)
$$\psi ^{m}$$: Mass fraction of each component in FW by city
$$\eta$$: Technology efficiency
T: Temperature ($$^{\circ }\hbox {C}$$)
$$\Omega _{j}^{sells}$$: Sales unitary price ($)
$$\Omega _{j}^{TippingFee}$$: Tipping fee unitary price ($)
$$f_{generated,j}$$: Generation emissions factor ($$\hbox {kg CO}_2$$ eq./MWh)
$$f_{avoid,i}$$: Avoided emissions factor ($$\hbox {kg CO}_2$$ eq./MWh)

## Sets

$$\Pi$$: Components
$$\lambda$$: Elements
$$\chi$$: Entity
$$\iota$$: WtE technologies
$$\tau$$: Time
$$\kappa$$: Waste type
$\mu$: Food waste types
